# Unraveling antibiotic resistance in *Achromobacter mucicolens* IA strain: genomic insights, structural analysis, and prospects for targeted therapeutics

**DOI:** 10.1128/spectrum.03926-23

**Published:** 2024-10-29

**Authors:** Sura Ali Al-Asadi, Batool Hazim Abdul Wahhab, Jamila Bootwala, Wifaq M. Ali Alwatar, Rusul Emaduldeen S. Al-Kahachi

**Affiliations:** 1 Department of Molecular and Medical Techniques, Biotechnology Research Centre, Al Nahrain University, Baghdad, Iraq; 2 Department of Microbiology, Faculty of Medicine, Mustansiriyah University, Baghdad, Iraq; 3 Genomics department, Genejenie, Mumbai, India; 4 Unit of Clinical and Communicable diseases, College of medicine, Baghdad University, Baghdad, Iraq; 5 Department of scholarships and cultural relationship, Republic of Iraq Ministry of Higher Education and Scientific Research, Baghdad, Iraq; NHLS Tygerberg/Stellenbosch University, Cape Town, Western Cape, South Africa

**Keywords:** *Achromobacter mucicolens IA strain*, antibiotic resistance, genomic analysis, efflux pumps, structural mutations

## Abstract

The mortality rate of infectious diseases caused by *Achromobacter mucicolens* is increasing. The enhanced antibiotic resistance among bacterial species through genetic transfer and mutations in the efflux mediating genes has made the treatment quite challenging. *A. mucicolens* is an aerobic, gram-negative, and non-fermenting opportunistic pathogen found in immunocompromised patients. *A. mucicolens* shows resistance against beta-lactams and other antibiotics through intrinsic resistance mechanisms, including multi-drug efflux pumps and beta-lactamases. In this study, the clinical isolate whole genome sequencing of *A. mucicolens* data was analyzed to identify the genes and mutations responsible for antimicrobial resistance. The identified genes and their mutants were then subjected to structural analysis to better understand the impact of mutations on the protein structure, and domain analysis was performed to investigate the role of domains in antibiotic resistance. A total of 4 genes, *acrR, macB, msbA,* and *tolC*, were identified with significant mutations, whereas *macB* was shortlisted for further analysis based on the conserved regions, sequence alignment, and the maximum number of mutations. All the mutants of the *macB* gene contain the two common domains, the ABC transporter-like ATP-binding domain and the AAA + ATPase domain. These domains are crucial in efflux mediating drug transport and can be targeted to design novel drugs for treating infections caused by *A. mucicolens*.

**IMPORTANCE:**

*Achromobacter* species represent a significant threat as opportunistic pathogens, particularly in healthcare settings. Their resilience to antibiotics, demonstrated by strains like A. *mucicolens*, poses a serious challenge in treating infections, especially in immunocompromised patients. This study emphasizes the critical need for heightened vigilance among healthcare professionals regarding *Achromobacter* infections. By analyzing the whole genome sequencing data of *A. mucicolens*, the study sheds light on the genetic basis of antimicrobial resistance, aiding in more targeted treatment strategies. Furthermore, structural and domain analyses offer insights into how mutations impact protein structure and function, crucial for developing effective interventions. Ultimately, implementing rigorous sanitation measures and antibiotic stewardship protocols is needed to mitigate the spread of *Achromobacter* and safeguard vulnerable patient populations.

## INTRODUCTION

*Achromobacter*, a rare pathogen, poses a significant threat in immunocompromised individuals, often leading to high mortality (1), reaching 20% by day 90 and rising to 27% in patients with three or more comorbidities, emphasizing the need for heightened attention to this pathogen (2).

*Achromobacter mucicolens* is a gram-negative, aerobic, and non-fermenting opportunistic bacteria that can be found in the intestinal tract of humans (3) and is ubiquitous and widely distributed in the environment (4). Infections caused by *A. mucicolens* include urinary tract infections, meningitis, osteomyelitis, abscesses, peritonitis, corneal ulcers, and pneumonia, specifically in immunocompromised patients (5). These infections majorly affect cystic fibrosis patients or patients with other immunity-related malignancies with a weak immune system (6).

*A. mucicolens* has been involved in drug resistance lately, making the treatment of infections challenging. Inherent resistance, a ubiquitous trait among bacterial species irrespective of prior antibiotic exposure, poses significant challenges in infection treatment. *A. mucicolens* exhibit intrinsic resistance mechanisms such as multi-drug efflux pumps and beta-lactamases (7).

Notably, the two multi-drug pumps are well-characterized, and many putative genes are responsible for the efflux pump (8). The AxyABM efflux pump genes of *Achromobacter* species significantly contribute to expelling antibiotics like cefepime, aztreonam, and cefuroxime. However, inhibiting this gene *in vitro* fails to heighten susceptibility to cephalosporins and aztreonam, suggesting that alternative mechanisms underpin *Achromobacter*’s drug resistance (9, 10).

The other efflux pump gene AxyXY-OprZ of *Achromobacter* bacteria, facilitates a broad antibiotic resistance spectrum, including cefepime, aminoglycosides, fluoroquinolones, carbapenems, and high-level resistance to aminoglycosides. Inhibiting AxyXY-OprZ *in vitro* restores sensitivity to tigecycline and aminoglycosides, indicating its significant contribution to antimicrobial resistance (11). Chromosomal beta-lactamase production from the gene *OXA-114 in Achromobacter species* results in resistance to penicillin G, ticarcillin, piperacillin, and some cephalosporins by hydrolyzing these antibiotics, particularly piperacillin while having a limited effect on cephalosporins (12).

Antimicrobial resistance efflux pump genes provide bacteria with mechanisms to withstand antibiotics, exacerbating difficulties in infection treatment. Factors such as spontaneous genetic alterations, pressure from antibiotic exposure, and horizontal gene transfer events lead to changes in their amino acid sequence, resulting in mutations in the proteins causing antimicrobial resistance through the efflux pump (13).

This study aimed to identify the mutated regions within the domains of the genes involved in antimicrobial resistance in the *A. mucicolens IA strain*. This study gives insights into the molecular and functional roles of several domains of different mutant models of *macB* protein implicated in antimicrobial resistance, which can contribute to future research by providing strategical targeting of the specific features of the *A. mucicolens*, ultimately leading to the development of effective therapeutics against *A. mucicolens*.

## MATERIALS AND METHODS

### Sample collection and preparation

The *A. mucicolens* strain used in this study was isolated from a sputum sample obtained from a 47-year-old patient with leukemia at the haemato-oncology department of Baghdad Teaching Hospital. The patient presented with a persistent cough and showed no response to various combinations of antibiotic drugs. The sputum sample was cultured on MacConkey agar plates to obtain pure cultures, which were subsequently utilized for identification and whole-genome sequencing. Genomic DNA was extracted from bacterial cells using the Presto Mini DNA Bacteria Kit (Geneaid Biotech Ltd., New Taipei City, Taiwan), followed by library preparation using the TruSeq DNA sample prep kit (Illumina, Inc., San Diego, CA, USA). The DNA was fragmented using ultrasonication, followed by adapter ligation and PCR enrichment processes.

The sample was obtained during routine clinical care, and the patient was not subjected to any additional interventions for the purposes of this work. Specifically, this work adheres to the institutional guidelines and policies.

### Genomic sequencing and identification of *Achromobacter* mucicolens strain

The whole genome sequencing was performed using sequencing-by-synthesis (SBS) technology, a next-generation sequencing (NGS) technology on NovaSeq 6000 platform on Illumina Inc. at Macrogen, South Korea. Sequencing generated 2.3 gigabases (Gb) of data with paired-end reads of read length of 150 base pairs (bp) at 395× coverage. The raw sequence reads generated were evaluated using FastQC v0.11.7 (Babraham Bioinformatics, Cambridge, UK) before and after trimming. Reads were trimmed (including adapter removal) using Trimmomatic v0.38 (Usadel Lab, Aachen, Germany) to discard sequences with a per-base sequence quality score. The short sequencing reads generated were reconstructed with *de novo* assembly using Unicycler v0.4.8 (University of Melbourne, Melbourne, Australia) and SPAdes v3.13.0 (Center for Algorithmic Biotechnology, St. Petersburg, Russia) assemblers and subjected to quality assessment using QUAST v5.2.0+galaxy1 (Center for Algorithmic Biotechnology, St. Petersburg, Russia). The assembly from Unicycler v0.4.8 (University of Melbourne, Melbourne, Australia) exhibited better quality with fewer contigs, larger contig sizes, higher N50 value, and cleaner assembly with no mismatches or N’s. The Unicylcer scaffold data were then run on CONTIGuator 2.7.4 (University of São Paulo, São Paulo, Brazil) to obtain structural insight and organization of the genomic sequence. Genome annotation employed Prokka 1.12 (Victorian Bioinformatics Consortium, Melbourne, Australia) and RAST 2.0 (The Fellowship for Interpretation of Genomes, Chicago, IL, USA) to obtain enhanced annotation comprehensiveness, validation, and functional insights by leveraging different databases (14). The genomic sequence of the *A. mucicolens* strain was deposited in GenBank under the BioProject number PRJNA224116, BioSample number SAMN21168694 with accession number CP082965. The SRA accession number is SRX11987443. The discriminatory online tool PubMLST (http://pubmlst.org/achromobacter [25]) (University of Oxford, Oxford, UK) utilized for multi-locus sequence typing (MLST) scheme focused on the single locus *nrdA* partial sequence (765 bp) for species-level identification present at locus start point position 751477 of our FASTA sequence. Identifying allele sequences or loci for selected housekeeping genes serves as a validating genetic marker, confirming the organism’s identification (15).

### SNP screening and clonality analysis and detection of drug-resistant genes

SNP screening was conducted on the genome sequence of the *A. mucicolens* strain, with a minimum coverage of 10×. This analysis identified putative SNPs occurring in over 50% of the read coverage. The determination of clonality involved calculating pairwise identity percentages and the number of SNPs between the sequenced sample and a reference sequence of *A. mucicolens*. Subsequently, the CARD database (v.3.1.4) (Comprehensive Antibiotic Resistance Database, McMaster University, Hamilton, Ontario, Canada) was utilized, along with the “*bwa*” software (Wellcome Sanger Institute, Hinxton, Cambridgeshire, UK), to ascertain the presence of drug-resistant genes in the *A. mucicolens* IA strain.

### Shortlisting of key survival and antibiotic-resistant genes

Bacterial growth and survival depend on several key genes that play crucial roles in defending against external fatal factors such as antibiotics and metals (16). Subsequently, selected genes were then taken from the resistant genes obtained from the CARD database (17) (https://card.mcmaster.ca/) in the previous step. CARD output is used to predict mutants coding DNA and mutant protein sequences of the original reference genes causing antibiotic resistance. The structural and functional features of protein sequences were analyzed further. However, for comparative purposes between *A. mucicolens* and the IA strain, the protein sequences of *A. mucicolens* were employed as reference standards.

### Multiple sequence alignment and conserved region analysis

To assess the conserved regions of the shortlisted proteins in *A. mucicolens*, multiple sequence alignment (MSA) involving shortlisted proteins between *A. mucicolens* and other *Achromobacter* species was performed. The shortlisted protein sequences were obtained from the UniProtKB database (18) (https://www.uniprot.org/) (UniProt Consortium, European Bioinformatics Institute, Hinxton, Cambridgeshire, UK), and the MSA was executed using Clustal Omega (19) (https://www.ebi.ac.uk/Tools/msa/clustalo/) (European Bioinformatics Institute, Hinxton, Cambridgeshire, UK). The species whose sequences were used for alignment are mentioned in Table 1. MEGAX (20) (https://www.megasoftware.net/) (Temple University, Philadelphia, PA, USA) was utilized to obtain the conserved region percentage of each shortlisted protein to obtain the most conserved protein among the aforementioned *Achromobacter* species. To assess the residue conserved regions rate among the species, the Scorecons server (21) (https://www.ebi.ac.uk/thornton-srv/databases/cgi-bin/valdar/scorecons_server.pl) was employed with the MSA from the previous step as input. Scorecons provides a score from 0 to 1, where 0 indicates no conserved regions, and 1 indicates a highly conserved residue. The residue conservation in a protein depends on the similarity between the new and the old amino acids and the probability of a substitution occurring. The protein with the most conserved residues was selected for further analysis.

**TABLE 1:** Species selected for multiple sequence alignment of *macB* and their accession IDs

Species	Accession IDs
*A. mucicolens*	A0A811GZB8
*A. deleyi*	A0A6S7BHU8
*A. kerstersii*	A0A6S7AFE0
*A. pestifer*	A0A7D4IJM7
*A. animicus*	A0A6S6ZV56
*A. piechaudii*	A0A6S7EAT8
*A. anxifer*	A0A6S7DN61
*A. insolitus*	A0A6S7FGX0
*A. spanius*	A0A3S9YQX0
*A. pulmonis*	A0A6S7DPD0
*A. spp*	A0A8G1TR51
*A. ruhlandi*i	A0A848NUN0
*A. deniificans*	A0A3R9FMH1
*A. spanius*	A0A3S4RYA6
*A. pestifer*	A0A67SA826
*A. agilis*	A0A446C7U7
*A. verterisilvae*	A0A446C6E3
*A. xylosydans*	A0A424WFF9
*A. spp*	A0A7X9W5Q0
*A. piechaudii*	D4X7Y3

### Protein domain analysis

Following the shortlisting of the protein based on conservation in MSA, protein domain analysis was conducted. The shortlisted protein is used as input in the InterPro database (22) (https://www.ebi.ac.uk/interpro/) (European Bioinformatics Institute, Hinxton, Cambridgeshire, UK) for protein domain analysis to identify the presence of domains and their regions in the protein. Similarly, the mutant protein sequences of a shortlisted protein were also analyzed on InterPro for the assessment of domain presence and their region spans. The domain sequences of the mutants were analyzed and compared in MSA using Clustal Omega to observe the conserved regions of the domains among the mutants.

### Comparative structure modeling

To observe the structural changes of a shortlisted protein, homology modeling, threading, and ab initio techniques were employed to obtain a model with the highest accuracy possible. MODELLER 10.3 (23) (https://salilab.org/modeller/) (University of California, San Francisco, CA, USA) was used to perform homology modeling by retrieving the best template from Protein Data Bank (Research Collaboratory for Structural Bioinformatics, Piscataway, NJ, USA). On the other hand, for threading, the IntFold (24) (https://www.reading.ac.uk/bioinf/IntFOLD/) (University of Reading, Reading, UK) server was used with the protein sequence as input for structure prediction using the threading technique. Similarly, for the ab initio modeling, a tool named OmegaFold (25) (https://cosmic-cryoem.org/tools/omegafold/) was used with the protein sequence as the input number of cycles 10.

To observe the structural changes in the mutant protein sequences of the shortlisted protein, the sequences retrieved from the CARD database were modeled into three-dimensional structures using homology modeling and ab initio techniques for the selection of the best model for each variant. MODELLER 10.3 was employed to perform homology modeling of each mutant separately with a template obtained from the Protein Data Bank. However, OmegaFold was used for ab initio modeling of the mutant protein sequences. Additionally, the individual domain sequences of mutant models were also predicted using OmegaFold. Furthermore, the ERRAT (26) (https://www.doe-mbi.ucla.edu/errat/) (University of California, Los Angeles, CA, USA), Verify3D (27) (https://www.doe-mbi.ucla.edu/verify3d/) (University of California, Los Angeles, CA, USA), and QMEAN (28) (https://swissmodel.expasy.org/qmean/) (Swiss Institute of Bioinformatics, Lausanne, Switzerland) tools were implemented to evaluate the highest accuracy of models of the predicted tertiary structures of the shortlisted reference protein and its respective mutant proteins.

### Comparative visualization of 3D structure features

The reference predicted model of the shortlisted protein was visualized using PyMOL (*The PyMOL Molecular Graphics System, Version 3.0 Schrödinger, LLC*) (https://pymol.org/2/) (Schrödinger, New York, NY, USA). The domains inside the model were annotated with different colors for indication. All the variants were superimposed onto the reference predicted model to assess structural changes gained or lost by the mutant models. The mutant models were also annotated based on the domain presence and region span. Moreover, the predicted individual domain structures were superimposed to visualize the structural changes in the individual domains.

## RESULTS

### Shortlisting of key survival and antibiotic-resistant genes

A list of four genes (*acrR, macB, msbA, and tolC*) was obtained by comparing the genes from the CARD database with the genes retrieved from the extensive literature review, which perform crucial roles in bacterial growth and survival, such as antibiotic efflux. Missense mutations in these genes (*acrR, macB, msbA, and tolC*) have been associated with antibiotic resistance, and mutations among these genes and their consequences were predicted by using the CARD database. The *macB* gene was predicted to have the highest number of mutations indicating its resistance to the macrolide antibiotic drug class, using the antibiotic efflux resistance mechanism, which belongs to the ATP-binding cassette (ABC) antibiotic efflux pump antimicrobial resistance (AMR) family. Furthermore, the *msbA* gene showed three mutations, whereas the *acrR* and *tolC* genes showed only one mutation. The genes and their number of mutations are mentioned in Table 2.

**TABLE 2:** Number of mutations identified in the shortlisted genes

Gene	Type	No. of mutations in a gene
*macB*	Macrolide export ATP-binding/permease protein MacB	39
*msbA*	Lipid A export ATP-binding/permease protein MsbA	3
*acrR*	HTH-type transcriptional regulator AcrR	1
*tolC*	Outer membrane protein	1

### Multiple sequence alignment and conserved region analysis

To evaluate the conserved regions of proteins in *A. mucicolens IA*, multiple sequence alignment (MSA) was conducted for each shortlisted protein (*acrR, macB, msbA,* and *tolC*) against corresponding proteins from other *Achromobacter* species. The MSA was performed for each of the shortlisted proteins to assess their conservation across *A. mucicolens IA* and other *Achromobacter* species. MEGA X was employed to obtain the overall conserved regions of the proteins among the *Achromobacter* species revealing that the *acrR* gene exhibits a 33% conserved region with 67% variability. In contrast, the *macB* gene displays a 75% conserved region with 25% variability. Similarly, the *msbA* gene demonstrates a 47% conserved region alongside 43% variability, and the *tolC* gene showcases a 71% conserved region with 29% variability. The shortlisted proteins and their respective conserved region ratios (%) are listed in Table 3.

**TABLE 3:** Conserved regions and variability of protein sequences among *Achromobacter* species after MSA

Gene	Conserved regions	Variability
*acrR*	33%	67%
*macB*	75%	25%
*msbA*	47%	43%
*tolC*	71%	29%

**Fig 1:**
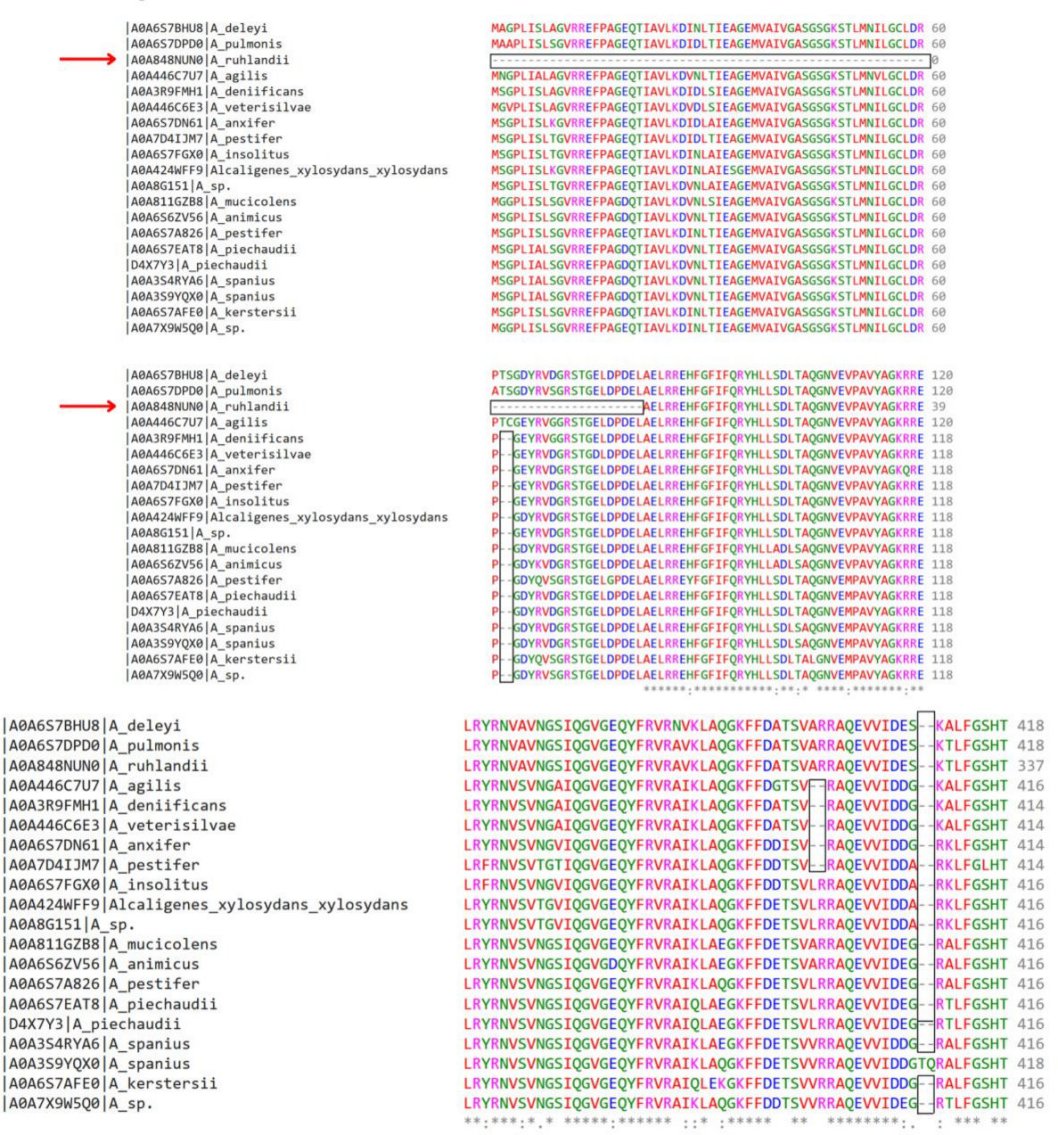
Multiple sequence alignment of MacB protein in 20 species of *Achromobacter*

Notably, the MSA revealed that the *macB* protein, compared with other shortlisted proteins, exhibited high conservation across 20 *Achromobacter* species. However, an initial gap of approximately 80 amino acids was noted in the *Achromobacter ruhlandii* species, indicated with a red arrow in Fig. 1. The multiple sequence alignment of 20 species of *Achromobacter* provided a better understanding of the conserved and variable regions of the *macB* protein sequence, as depicted in Fig. 1.

### Protein domain analysis

The “InterPro” database was used to identify the presence of domains and their regions in the *macB* protein of *Achromobacter mucicloens*. It was found that the *macB* protein showed four domains, including the ABC transporter-like ATP-binding domain, AAA + ATPase, MacB-PCD, and ABC3 permease domain, whereas the ABC transporter-like ATP-binding domain was identified as the longest.

Similarly, each mutant protein sequence of the *macB* protein in the *A. mucicloens IA* strain was also analyzed on InterPro to assess domain presence and their region spans. It was revealed that two domains of *macB* protein (ABC transporter-like ATP-binding domain and AAA + ATPase) were commonly present among all the mutants of *macB* except for the mutant model of “Sequence 26.” Notably, the mutant model of “Sequence 36” showed all four domains of the *macB* protein. Last, some mutants had different domain spans. The similar domains identified in different mutants are depicted in Table 4 All the details regarding the length of mutants and the number of domains present in them are provided in the supplementary file.

**TABLE 4:** Reference and mutant proteins and their domain analysis

Protein/Mutants	Domains
Reference *macB*	ABC transporter-like ATP-binding domain AAA + ATPase MacB-PCD ABC3 permease domain
Mutant 26	MacB-PCD ABC3 permease domain
Mutant 36	ABC transporter-like ATP-binding domain AAA + ATPase MacB-PCD ABC3 permease domain
All remaining mutants	ABC transporter-like ATP-binding domain AAA + ATPase

### Comparative visualization of 3D structure features

A comparative three-dimensional conformation prediction of the *macB* reference and its mutants was employed using MODELLER, OmegaFold, and IntFold. The *macB* protein was used as a template that was retrieved from the Protein Data Bank (PDB ID: 5WS4) for the homology modeling through MODELLER. It was observed that the reference protein predicted through OmegaFold had much better accuracy than the models predicted using MODELLER and IntFold. For each mutant, a model was predicted using MODELLER, OmegaFold. The structure evaluation for the mutant structures revealed that some models had higher accuracy when predicted using OmegaFold compared with MODELLER. Therefore, the structures that passed the evaluation thresholds were selected for further analysis for each mutant. Furthermore, the ERRAT scores and QMEANDisCo Global scores ranged from 87.6 to 99.41 and 0.61 to 0.82, respectively. The ERRAT score of 87.6 was shown by mutant model 17, whereas the ERRAT score of 99.41 was shown by mutant model 18. Similarly, the QMEANDisCo Global score of 0.61 was shown by mutant model 5, whereas 0.82 was shown by four mutant models, including models 2,32,34, and 38. Finally, the mutant model for 26 failed the verification test based on the scores depicted in Table 5.

**TABLE 5:** OmegaFold models evaluation and RMSD values of superimposition

Model name	ERRAT score	Verify3D score	QMEANDisCo global score	RMSD value
Reference *macB* (1)	91.4286	Pass	0.76	--
2	97.4249	Pass	0.82	1.026
3	94.14	Pass	0.65	1.078
4	98.75	Pass	0.80.	1.099
5	88.3	Pass	0.61	1.256
6	95.81	Pass	0.67	0.899
7	93.83	Pass	0.65	0.991
8	94.7195	Pass	0.63	0.959
9	95.0758	Pass	0.65	1.026
10	97.6	Pass	0.80.	1.152
11	90.12	Pass	0.67	1.01
12	90.18	Pass	0.64	0.998
13	99.13	Pass	0.67	0.999
14	93.8843	Pass	0.62	0.93
15	93.57	Pass	0.65	0.911
16	98.73	Pass	0.81	0.954
17	87.6	Pass	0.66	1.037
18	99.41	Pass	0.75	1.248
19	98.71	Pass	0.81	1.061
20	94.28	Pass	0.67	1.077
21	98.77	Pass	0.80.	1.034
22	94.8	Pass	0.66	1.022
23	94.18	Pass	0.76	1.087
24	95.41	Pass	0.64	0.986
25	94.71	Pass	0.70.	1.093
26	90.36	Fail	0.64	1.036
27	90.14	Pass	0.64	1.177
28	96.63	Pass	0.81	0.92
29	93.3	Pass	0.65	1.012
30	97.08	Pass	0.76	1.079
31	94	Pass	0.65	1.04
32	97.03	Pass	0.82	1.01
33	98.59	Pass	0.72	0.873
34	99.14	Pass	0.82	0.978
36	91.9048	Pass	0.76	0.097
37	92.068	Pass	0.70.	0.985
38	98.29	Pass	0.82	0.985
39	93.15	Pass	0.65	1.097

**Fig 2:**
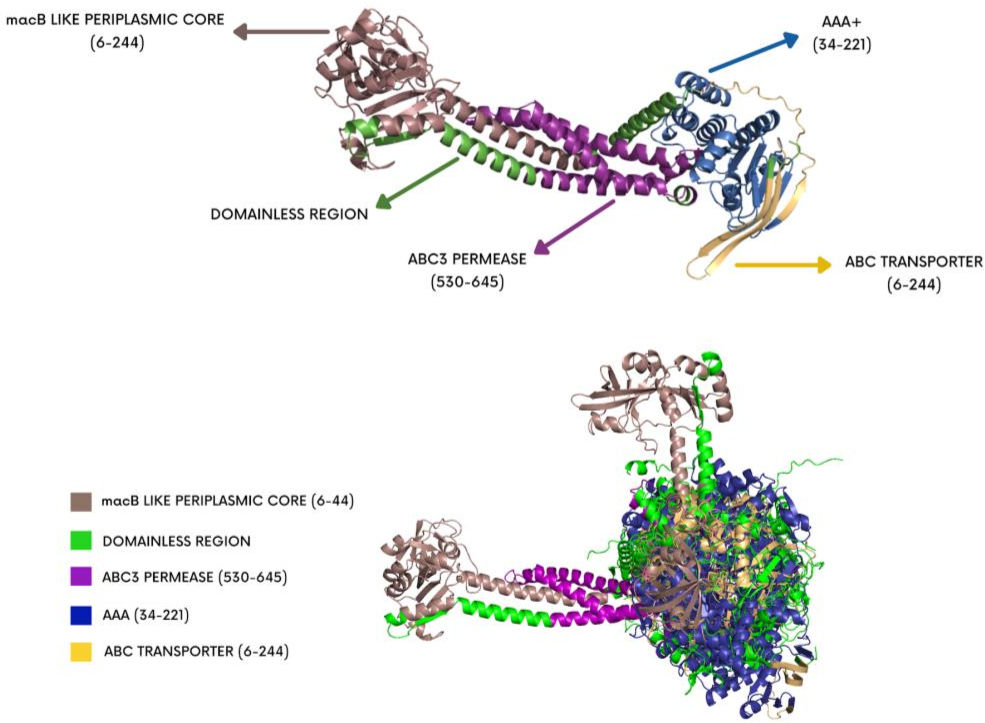
3D representation of the domains present in MacB protein.

**Fig 3:**
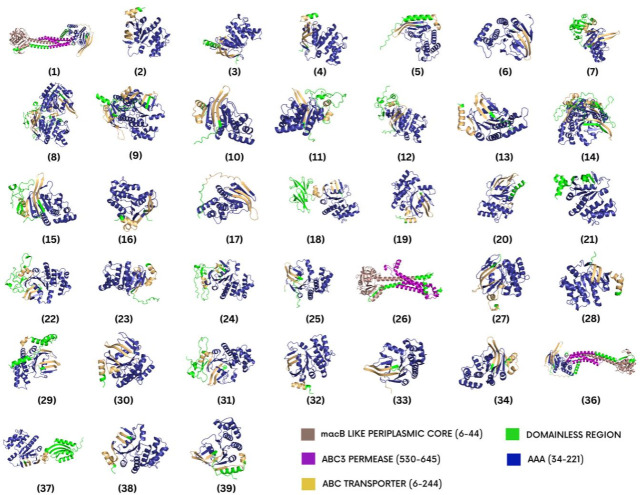
Structures of antibiotic resistance causing mutants of MacB protein.

The reference predicted model of the *macB* protein was visualized using PyMOL. The domains inside the model were annotated with different colors for indication as shown in Fig. 2. All the variants were superimposed onto the reference predicted model to assess structural changes gained or lost by the mutant models. Moreover, the Root Mean Square Deviation (RMSD) values of superimposed models ranged from 0.097 to 1.256. The RMSD value of 0.097 was observed for model 36, whereas the RMSD value of 1.256 was observed for model 5. The mutant models were also annotated based on the domain presence and region span illustrated in Fig. 3. The RMSD values of each superimposed mutant have been mentioned in Table 5.

**Fig 4:**
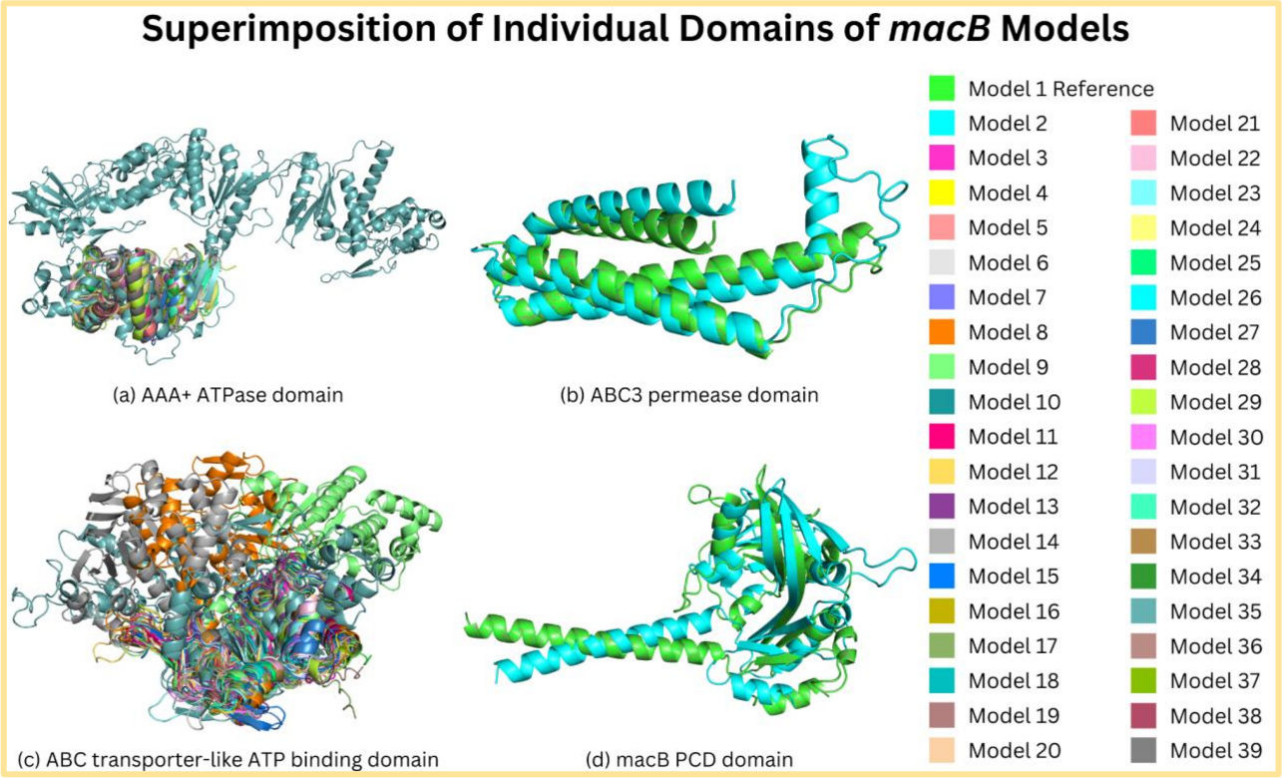
Individual domains of macB models are superimposed to show the structural changes due to mutations. (**A**) AAA + ATPase domain was present in all macB models except for the mutant model 26. (**B**) ABC3 permease domain was found in mutant models 26 and 36, and the reference model. (**C**) ABC transporter-like ATP binding domain was present in all macB models except for the mutant model 26. (**D**) MacB-PCD domain was present in the mutant models 26 and 36 and the reference model.

Moreover, the 3D structure prediction of the individual domains was performed using OmegaFold, resulting in the structures of the domains (ABC transporter-like ATP-binding domain, AAA + ATPase, MacB-PCD, and ABC3 permease domain) that were superimposed using PyMOL. The superimposition of the individual domains showed the structural differences between the individual domain structures due to the effects of the mutations. The ABC transporter-like ATP-binding domain and AAA + ATPase domain were found to be present in all *macB* models except for the mutant “Mutant 26.” Nonetheless, the MacB-PCD domain and ABC3 permease domain were found to be present in mutant “Mutant 26,” “Mutant 36,” and reference models. The superimposition of the individual domains of *macB* models is shown in Fig. 4.

## DISCUSSION

This study analyzed the clinical isolate of the *A. mucicolens* IA strain’s whole genome sequencing data to identify the genes responsible for increased antimicrobial resistance. The *acrR, macB, msbA, and tolC* genes were determined by combining the output of sequencing data analysis and the literature review, including all the genes playing a crucial role in antimicrobial resistance (29–31). Among these *acrR, macB, msbA, and tolC* genes, the highest number of 39 mutations were identified in *macB*; three mutations were found in *msbA*, whereas the remaining *acrR* and *tolC* genes showed one mutation. Next, multiple sequence alignment of each gene (*acrR*, *macB*, *msbA*, and *tolC*) among different species of *Acromobacter* was performed using Clustal Omega and MEGAX to better understand the mutations and the conserved regions of the sequence. Comparative with the other genes (*acrR*, *msbA*, and *tolC*), the maximum conserved region was shown by *macB* as a conserved region percentage of 75%, and a total of 25% region was found to be semi-conserved. Based on the number of 39 mutations in *macB* and findings of the conserved regions through multiple sequence alignment, the *macB* gene was selected for further structural analysis of its protein. Moreover, comparative analysis of the predicted mutant *macB* and its reference model structures indicated different RMSD values when superimposed on its reference *macB* protein, revealing the impact of mutations on the structures of *macB* protein. Domain analysis of *macB* and mutants revealed that two domains, ABC transporter-like ATP-binding domain and AAA + ATPase domain, were present commonly in all mutations except the mutant “Mutant 26,” which had the other two domains, MacB-PCD and ABC3 permease-domain. Furthermore, the superimposition of the individual domain structures of all mutants indicated the structural changes among different mutants due to mutations, suggesting the role of each domain in antimicrobial resistance. However, the two significant domains, the ABC transporter-like ATP-binding domain and AAA + ATPase domain within the *macB* protein, are the focus of research against antimicrobial resistance.

ABC transporter-like ATP-binding domain of *macB* protein hydrolyzes ATP to release energy in different biological processes. This domain plays an important role in virulence and bacterial pathogenesis by contributing to multi-drug resistance (32). Major functions of ABC transporter-like ATP-binding domain include the import and export of different cellular substrates. It aids in translocating a wide range of molecules like toxins, metals, proteins, and xenobiotics outside the cytosol. The *tolC*, along with *macB*, forms a tripartite pump responsible for expelling antibiotics from bacterial cells. However, in addition to its role in this pump, some *macB* proteins function independently in diverse physiological processes such as cell division, antibiotic sensing, and lipoprotein trafficking (29). The tripartite efflux machinery or pumps of *macB* are, therefore, influential in the survival of bacteria, specifically during pathogenic infections.

The other significant domain of *macB* protein, AAA + ATPase, is present in all living organisms regulating diverse cellular processes (33). This domain contains a structurally conserved central subdomain called ATPase, AAA + module, of about 250 amino acids. Other subdomains of AAA + ATPase include the N-terminal P-loop NTPase α-β-α subdomain connected to a relatively smaller C-terminal all-α subdomain (34), suggesting that the ATP hydrolysis activity of this domain allows the enhanced efflux of antibiotics leading to antimicrobial resistance. Both of these domains of *macB* exhibit ATP hydrolysis activity, which allows drug transport through antiporters.

The majority of the mutations identified within these two domains imply that these regions have more to do with antibiotic resistance and increased pathogenicity through efflux (35, 36). It enhances the efficiency of the tripartite efflux pump and increases resistance against antimicrobial drugs as mutants contain only these two domains critical for the efflux of antibiotics (37, 38). Mutations can increase the expression of the efflux pump proteins, leading to enhanced antibiotic resistance (39, 40) and the survival of bacteria within their host body.

Hence, in view of the results, it is indicated that the two domains, ABC transporter-like ATP-binding domain and AAA + ATPase domain, are the pivotal components of *macB* protein in its pathogenicity and antimicrobial resistance as these domains are consistently present in all mutants of *macB* protein except for one. Furthermore, the known functions of these domains encompass a range of processes, including the import and export of different cellular substrates and regulating diverse cellular processes, but more importantly, are involved in antimicrobial resistance and increased pathogenicity. Consequently, targeting these specific domains of *macB* protein holds significant promise for inhibiting *A. mucicolens*. This insight provides a foundation for the development of novel therapeutics aimed at disrupting these critical domains, ultimately leading to *A. mucicolens* inhibition.

### Conclusion

Using the whole genome sequencing data analysis of the clinical isolate of *A. mucicolens,* we identified genes and mutations that can be used as targets to design novel drugs that can reduce antimicrobial resistance. Four genes, *acrR, macB, msbA, and tolC,* were filtered from the list of genes that play a significant role in survival and antimicrobial resistance. The gene *macB* showed a maximum number of mutations and highly conserved regions; hence, it was selected for the structural analysis and comparative visualization of its mutants. Two important ATP binding domains, the ABC transporter-like ATP-binding domain and the AAA + ATPase domain, were found in all the mutants except for one mutant, implying that these play a crucial role in increased antimicrobial resistance and pathogenicity. Targeting mutations in these domains can be a novel approach to designing drugs to combat infections caused by *A. mucicolens*.

## Data Availability

The genomic sequence of the *A. mucicolens* strain *IA* was deposited in GenBank under BioProject number PRJNA224116, BioSample number SAMN21168694, with accession number CP082965, revised version NZ_CP082965.1. The SRA accession number is SRX11987443. This strain was sequenced using Illumina NovaSeq 6000 technology. Despite initially being identified as *Achromobacter denitrificans* through biochemical testing using the Vitek two system, further analysis via whole-genome sequencing and multilocus sequence typing (MLST) of the *nrdA* gene (765 bp sequence) confirmed its classification as *A. mucicolens*.

## SUPPLEMENTAL MATERIAL

Table S1 spectrum.03926-23-s0001.docx Reference and mutant proteins and their domain analysis.

ASM does not own the copyrights to Supplemental Material that may be linked to, or accessed through, an article. The authors have granted ASM a non-exclusive, world-wide license to publish the Supplemental Material files. Please contact the corresponding author directly for reuse.
