# Supplementary material for: Unraveling antibiotic resistance in Achromobacter mucicolens IA strain: genomic insights, structural analysis, and prospects for targeted therapeutics
Source: Microbiol Spectr. 2024 Oct 29;12(12):e03926-23. doi: 10.1128/spectrum.03926-23 (PMC11619425; doi:10.1128/spectrum.03926-23)
Supplement: Table S1 — Reference and mutant proteins and their domain analysis. [file spectrum.03926-23-s0001.docx]

Supplemental Table 1: Reference and Mutant Proteins and their Domain Analysis

|  | **Length** | **Domains** |
| --- | --- | --- |
| Reference Protein | 652 | 1. ABC_transporter-like-ATP-binding domain (6-244)  2. AAA+_ATPase (34-221)  3. MacB-PCD (275-494)  4. ABC3 - permease - dom (530-645) |
| Mutant 2 | 241 | 1. ABC_transporter-like-ATP-binding domain (2-236)  2. AAA+_ATPase (26-213) |
| Mutant 3 | 264 | 1. ABC_transporter-like-ATP-binding domain (6-241)  2. AAA+_ATPase (36-218) |
| Mutant 4 | 249 | 1. ABC_transporter-like-ATP-binding domain (6-245)  2. AAA+_ATPase (35-230) |
| Mutant 5 | 273 | 1. ABC_transporter-like-ATP-binding domain (27-254)  2. AAA+_ATPase (51-232) |
| Mutant 6 | 228 | 1. ABC_transporter-like-ATP-binding domain (2-228)  2. AAA+_ATPase (27-218) |
| Mutant 7 | 367 | 1. ABC_transporter-like-ATP-binding domain (24-263)  2. AAA+_ATPase (52-239) |
| Mutant 8 | 617 | 1. ABC_transporter-like-ATP-binding domain  (316-567)  (15-269)  2. AAA+_ATPase  (351-543)  (41-246) |
| Mutant 9 | 550 | 1. ABC_transporter-like-ATP-binding domain  (7-259)  (289-528)  2. AAA+_ATPase  (35-236)  (314-505) |
| Mutant 10 | 262 | 1. ABC_transporter-like-ATP-binding domain (11-250)  2. AAA+_ATPase (35-227) |
| Mutant 11 | 333 | 1. ABC_transporter-like-ATP-binding domain (10-260)  2. AAA+_ATPase (38-237) |
| Mutant 12 | 340 | 1. ABC_transporter-like-ATP-binding domain (12-260)  2. AAA+_ATPase (45-252) |
| Mutant 13 | 240 | 1. ABC_transporter-like-ATP-binding domain (8-236)  2. AAA+_ATPase (32-213) |
| Mutant 14 | 628 | 1. ABC_transporter-like-ATP-binding domain  (16-272)  (333-572)  2. AAA+_ATPase  (44-249)  (357-549) |
| Mutant 15 | 335 | 1. ABC_transporter-like-ATP-binding domain (12-266)  2. AAA+_ATPase (38-243) |
| Mutant 16 | 246 | 1. ABC_transporter-like-ATP-binding domain (6-241)  2. AAA+_ATPase (30-218) |
| Mutant 17 | 251 | 1. ABC_transporter-like-ATP-binding domain (2-237)  2. AAA+_ATPase (27-214) |
| Mutant 18 | 351 | 1. ABC_transporter-like-ATP-binding domain (4-234)  2. AAA+_ATPase (28-211) |
| Mutant 19 | 245 | 1. ABC_transporter-like-ATP-binding domain (4-238)  2. AAA+_ATPase (28-215) |
| Mutant 20 | 256 | 1. ABC_transporter-like-ATP-binding domain (2-234)  2. AAA+_ATPase (30-211) |
| Mutant 21 | 252 | 1. ABC_transporter-like-ATP-binding domain (10-244)  2. AAA+_ATPase (34-221) |
| Mutant 22 | 336 | 1. ABC_transporter-like-ATP-binding domain (17-267)  2. AAA+_ATPase (45-244) |
| Mutant 23 | 267 | 1. ABC_transporter-like-ATP-binding domain (19-263)  2. AAA+_ATPase (43-240) |
| Mutant 24 | 337 | 1. ABC_transporter-like-ATP-binding domain (14-264)  2. AAA+_ATPase (42-249) |
| Mutant 25 | 237 | 1. ABC_transporter-like-ATP-binding domain (17-235)  2. AAA+_ATPase (45-233) |
| Mutant 26 | 426 | 1. MacB-PCD (38-255)  2. ABC3 - permease - dom (286-419) |
| Mutant 27 | 256 | 1. ABC_transporter-like-ATP-binding domain (5-253)  2. AAA+_ATPase (30-230) |
| Mutant 28 | 246 | 1. ABC_transporter-like-ATP-binding domain (6-241)  2. AAA+_ATPase (30-218) |
| Mutant 29 | 294 | 1. ABC_transporter-like-ATP-binding domain (5-239)  2. AAA+_ATPase (33-216) |
| Mutant 30 | 250 | 1. ABC_transporter-like-ATP-binding domain (2-242)  2. AAA+_ATPase (26-219) |
| Mutant 31 | 329 | 1. ABC_transporter-like-ATP-binding domain (8-258)  2. AAA+_ATPase (36-235) |
| Mutant 32 | 244 | 1. ABC_transporter-like-ATP-binding domain (2-236)  2. AAA+_ATPase (26-221) |
| Mutant 33 | 225 | 1. ABC_transporter-like-ATP-binding domain (7-224)  2. AAA+_ATPase (35-220) |
| Mutant 34 | 242 | 1. ABC_transporter-like-ATP-binding domain (4-238)  2. AAA+_ATPase (28-214) |
| Mutant 35 | 1931 | 1. ABC_transporter-like-ATP-binding domain  (375-633)  (653-995)  2. AAA+_ATPase  (677-964)  (1082-1536)  (1604-1891) |
| Mutant 36 | 652 | 1. ABC_transporter-like-ATP-binding domain (6-244)  2. AAA+_ATPase (34-221)  3. MacB-PCD (275-494)  4. ABC3 - permease - dom (530-645) |
| Mutant 37 | 362 | 1. ABC_transporter-like-ATP-binding domain (2-241)  2. AAA+_ATPase (30-218) |
| Mutant 38 | 243 | 1. ABC_transporter-like-ATP-binding domain (2-236)  2. AAA+_ATPase (26-213) |
| Mutant 39 | 272 | 1. ABC_transporter-like-ATP-binding domain (18-249)  2. AAA+_ATPase (46-227) |

# 
